# Role of the kidneys in the redistribution of heme-derived iron during neonatal hemolysis in mice

**DOI:** 10.1038/s41598-019-47414-y

**Published:** 2019-07-31

**Authors:** Aleksandra Bednarz, Paweł Lipiński, Rafał R. Starzyński, Mateusz Tomczyk, Witold Nowak, Olga Mucha, Mateusz Ogórek, Olga Pierzchała, Aneta Jończy, Robert Staroń, Julia Śmierzchalska, Zenon Rajfur, Zbigniew Baster, Alicja Józkowicz, Małgorzata Lenartowicz

**Affiliations:** 1 Department of Genetics and Evolution, Institute of Zoology and Biomedical Research, Jagiellonian University, Gronostajowa 9, 30-387 Kraków, Poland; 2 Department of Molecular Biology, Institute of Genetics and Animal Breeding, Polish Academy of Sciences, 05-552 Magdalenka, Jastrzębiec Poland; 3 Department of Medical Biotechnology, Faculty of Biochemistry, Biophysics and Biotechnology, Jagiellonian University, Gronostajowa 7, 30-387 Kraków, Poland; 4 Department of Molecular and Interfacial Biophysics, Faculty of Physics, Astronomy and Applied Computer Science, Jagiellonian University, Łojasiewicza 11, 30-348 Kraków, Poland

**Keywords:** Nephrons, Transport carrier

## Abstract

Moderate intravascular hemolysis is a common condition in newborns. It is followed by the accumulation of bilirubin, which is a secondary product of the activity of heme oxygenase-1, an enzyme that catalyzes the breakdown of heme released from disrupted erythrocytes and taken up by hepatic macrophages. Although these cells are a major site of enzymatic heme breakdown in adults, we show here that epithelial cells of proximal tubules in the kidneys perform the functions of both heme uptake and catabolism in mouse neonates. A time-course study examining mouse pups during the neonatal period showed a gradual recovery from hemolysis, and concomitant decreases in the expression of heme-related genes and non-heme iron transporters in the proximal tubules. By adjusting the expression of iron-handling proteins in response to the disappearance of hemolysis in mouse neonates, the kidneys may play a role in the detoxification of iron and contribute to its recirculation from the primary urine to the blood.

## Introduction

In humans, neonatal jaundice is one of the most common pathophysiological conditions, affecting all pre-term and a high proportion of term newborns. It is usually due to excessive bilirubin production and/or impaired conjugation, resulting in an increased bilirubin concentration. Generally, this is an entirely benign process that is resolved by the end of the first week of life without treatment^1^. Mild intravascular hemolysis is often the primary cause of hyperbilirubinemia during the first week of life and is a feature of normal newborn physiology^2^. The inevitable nature of neonatal hemolysis is mainly due to the increased red blood cell (RBC) turnover rate in newborns, which is up to 2–3 times that of a normal adult^3^. The reduced neonatal RBC lifespan is thought to be caused by the increased oxidant sensitivity of these cells and relative instability of fetal hemoglobin, which may entail RBC membrane damage^4^. The disruption of fetal erythrocytes is associated with the release to the bloodstream of highly reactive pro-oxidants such as free hemoglobin (Hb) and its porphyrin component, heme. Under conditions of limited hemolysis, the potentially deleterious effects of Hb and heme are efficiently counteracted by protective systems composed of specific ligands (haptoglobin and hemopexin, respectively) and receptors (CD163 and CD91, respectively) that lead to Hb and heme binding, neutralization and clearance, mainly by macrophages of the reticuloendothelial system and hepatocytes^5^. Subsequent detoxification of heme released from Hb in the cellular compartment involves the activity of heme oxygenase 1 (HO1), a key enzyme in heme catabolism that cleaves the porphyrin ring, at the expense of molecular oxygen, to release ferrous iron, CO and biliverdin^6^. Freed heme iron is then neutralized by ferritin, a multimeric cytosolic iron-storage protein, or recycled to the circulation *via* ferroportin (Fpn), the sole known iron exporter, while biliverdin is rapidly enzymatically reduced to bilirubin^7^. Hemolysis-dependent overproduction of bilirubin during the neonatal period, in combination with its defective metabolism through glucuronidation by developmentally immature UDP-glucuronosyltransferase, reduces bilirubin excretion into the bile and predisposes newborns to high total serum bilirubin levels^8^.

In many hemolytic disorders, excessive hemolysis may overwhelm endogenous plasma haptoglobin/hemopexin and other scavenging mechanisms as well as heme degradation pathways in hepatic, splenic and bone marrow macrophages. Human^9^ and animal^10,11^ studies have clearly shown that under severe hemolytic conditions, the kidneys participate in the management of heme and non-heme iron released from disrupted RBCs. There is growing evidence that renal tubular cells can adapt to exposure to high levels of heme by inducing HO1 and ferritin^9,10^. Furthermore, it has been established that these polarized epithelial cells are equipped with a competent molecular machinery that allows iron uptake from the primary urine, its transport across the apical membrane and finally its redistribution through the basolateral membrane into the circulation^12–14^.

Most studies on mouse neonatal jaundice have been performed on genetically modified animals showing impaired bilirubin conjugation and hyperbilirubinemia, which consequently experience early neonatal lethality due to bilirubin toxicity^15^. Here, our aim was to explore the role of the kidneys in iron handling during naturally occurring neonatal jaundice in mice. We wished to determine whether moderate physiological hemolysis in mouse neonates affects iron metabolism in the kidneys. This is of particular importance in the context of the developmental delay in early postnatal hepatic iron management^16^. In humans, the knowledge about the involvement of kidneys in neonatal iron metabolism could be also very useful in the treatment of severe cases of neonatal jaundice especially in newborns with congenital nephropathies. Our results revealed a gradual disappearance of hemolysis in mice between days 3 and 11 after birth, a phenomenon closely correlated with the orchestrated decrease in the expression of HO1 and proteins responsible for apical/basolateral transport of heme and non-heme iron in the epithelial cells of kidney proximal tubules. We hypothesize that initially (during the first week of life), the presence of high levels of iron-handling proteins in the kidneys in response to hemolysis, not only reduces iron toxicity but also contributes to the economical use of this microelement in mouse neonates.

## Results

### Hyperbilirubinemia and evidence of intravascular hemolysis in neonatal mice

**Figure 1:**
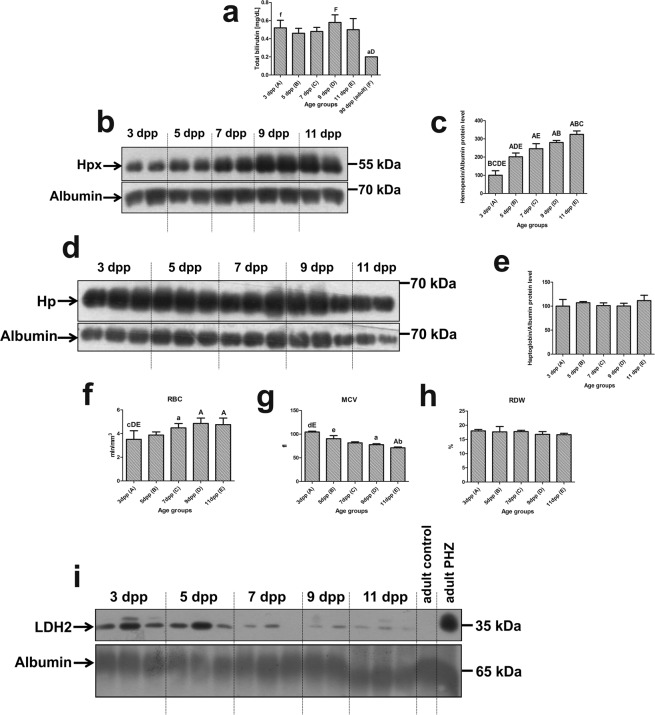
Markers of hemolysis in the plasma of mouse neonates. (**a**) Total bilirubin concentration in the plasma of mouse neonates and adult (3-month-old) mice. Values are expressed as the means ± S.D. N values for each group are: 3dpp = 5, 5dpp = 5, 7dpp = 5, 9dpp = 5, 11dpp = 5, 90dpp = 5. Data set is not normally distributed, therefore, nonparametric, Kruskal-Wallis ANOVA was used (p = 0,004, df = 5, H = 17,3). Dunn’s Multiple Comparison Test was used as post-hoc test. Plasma levels of hemopexin (Hpx) (**b**) and haptoglobin (Hp) (**d**) assessed by Western blotting. The blots were reprobed with polyclonal anti-albumin antibody as a loading control. Results come from 2 separate blots and relative levels of proteins were standardized to a percentage with %S.D. in order to eliminate technical differences between blots. Immunolabeled Hpx (**c**) and Hp (**e**) and albumin control bands were quantified using a Molecular Imager and the relative levels of the test proteins (means ± S.D.) are plotted in arbitrary units. N values in each group for both Hp and Hpx are: 3dpp = 5, 5dpp = 5, 7dpp = 5, 9dpp = 5, 11dpp = 4. Data set for Hpx has normal distribution, therefore, one-way ANOVA was used (p < 0.0001, df = 4, F = 51,40). Tukey’s Multiple Comparison Test was used as post-hoc test. Data set for Hp is not normally distributed, therefore, Kruskal-Wallis ANOVA was used (p = 0,9622, df = 4, H = 0,608) but no statistically significant differences were observed. Capital and small letters over bars in the charts (**a**,**c**,**e**) denote significant differences between age groups, with p < 0.01 and p < 0.05, respectively. dpp – days postpartum. (**f**–**h**) RBC indices potentially indicating the occurrence of hemolysis: (**f**) RBC- Red blood cells count, (**g**) MCV - mean corpuscular volume of a red blood cell, (**h**) RDW - red cell distribution width. Values are expressed as the means ± S.D. N values for each group are: 3dpp = 6, 5dpp = 5, 7dpp = 5, 9dpp = 4, 11dpp = 7. Data set for RBC and RDW has normal distribution, therefore, one-way ANOVA was used and Tukey’s Multiple Comparison Test was used as post-hoc test for the RBC and post test for linear trend (p = 0,0097) was used for RDW. Data set for MCV is not normally distributed, therefore, nonparametric, Kruskal-Wallis ANOVA was used. Dunn’s Multiple Comparison Test was used as post-hoc test. Capital and small letters over bars in the charts (**a**,**c**,**f**,**g**,**h**) denote significant differences between age groups, with p < 0.01 and p < 0.05, respectively. dpp – days postpartum. (**i**) Lactate dehydrogenase (LDH2) plasma levels in the serum from all experimental group of neonates analyzed by Western blotting as described in Materials and Methods. Lack of LDH2-positive band in the serum of the adult, intact mouse. Prominent immunopositive band is visible in the serum from adult phenylhydrazine-treated (PHZ) mouse (positive control). The blots were reprobed with polyclonal anti-albumin antibody as a loading control.

The concentration of bilirubin in the blood plasma of mice was measured during the neonatal period from day 3 to day 11 after birth and in 90-day-old adult mice. Serum bilirubin was more than 2-fold elevated in the neonates compared with adults (Fig. 1a). Postnatal instability of fetal erythrocytes and the increased risk of developing intravascular hemolysis is usually the initial cause of hyperbilirubinemia during the first week of life^2^. We therefore studied the hallmarks of intravascular hemolysis in mouse neonates, such as hemopexin and haptoglobin (heme and hemoglobin scavenging proteins, respectively). We also evaluated RBC indices potentially indicating the occurrence of hemolysis. Under conditions of increased leakage of heme and/or hemoglobin into the blood, serum levels of both proteins are generally low^5^. We detected an increase in hemopexin in the serum of mouse neonates, with values measured on days 3 and 11 postpartum differing by more than 3-fold (Fig. 1b,c). In contrast, serum haptoglobin levels remained unchanged throughout the entire neonatal period (Fig. 1d,e). Among mammals, the mouse is unique because there is no strict requirement for haptoglobin as a ligand for hemoglobin scavenging from the circulation^17^. As regards RBC parameters, we showed that the RBC count is gradually increased throughout the experimental period (Fig. 1f) while mean corpuscular volume (MCV) is linearly decreased (Fig. 1g) and RDW values show tendency to decrease (Fig. 1h). Collectively, these data suggest elimination from the circulation of fetal RBC characterized by greater cell volume^18^. It is also confirmed by the results showing that the lactate dehydrogenase (LDH2) plasma levels (used as a marker for intravascular hemolysis in neonates^19^ is high in the 3- and 5-day-old mice and next decreased with the age of neonates. It indicates on gradual disappearance of hemolysis in mouse neonates between days 3 and 11 after birth (Fig. 1i).

### Increase in *Hmox1* gene expression and disappearance of erythroblastic islands in the early postnatal liver

**Figure 2:**
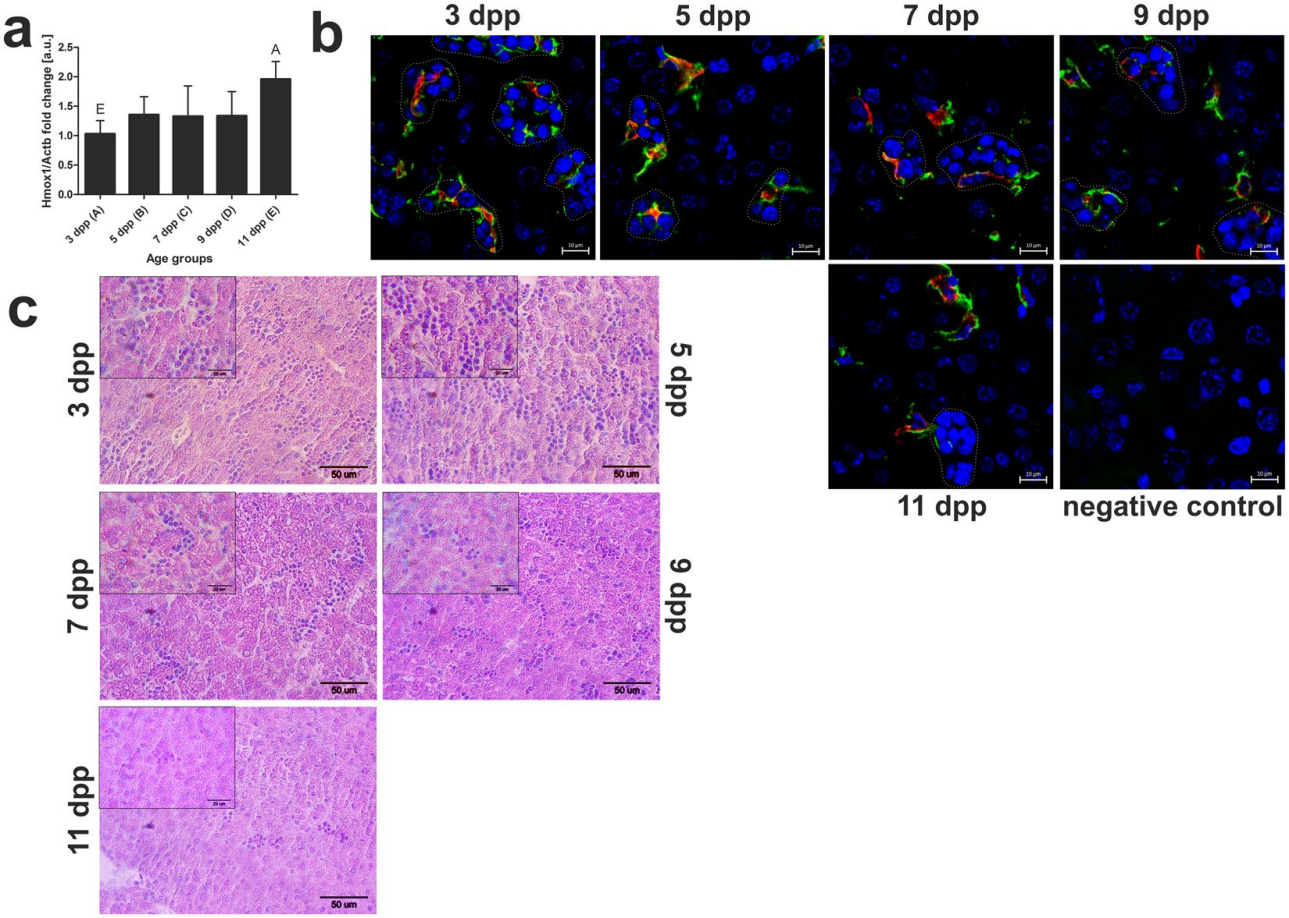
Increase in *Hmox1* gene expression and disappearance of erythroblastic islands in the liver of mice during the neonatal period. (**a**) RT-qPCR analysis of HO1 transcript levels in the liver of mouse neonates from 3 to 11 days old. The histogram displays the relative HO1 mRNA levels in arbitrary units (means ± S.D.). N values for each group are: 3dpp = 5, 5dpp = 5, 7dpp = 4, 9dpp = 4, 11dpp = 5. Data set for *Hmox1* expression has normal distribution, therefore, one-way ANOVA was used (p = 0,0098, df = 4, F = 4, 6). Tukey’s Multiple Comparison Test was used as post-hoc test. Capital letters denote significant differences between the age groups, with p < 0.01. (**b**) Immunofluorescent staining of HO1 (red channel) and co-localization of HO1 with F4/80, a macrophage marker (green channel) in the livers of neonatal mice, analyzed by confocal microscopy. Hematopoiesis centers in the liver are surrounded by dotted white lines. Cell nuclei were counterstained with DAPI (blue). Scale bars correspond to 10 µm. To confirm the specificity of HO1 and F4/80 detection, liver sections were incubated only with the mixed secondary antibodies (negative control). (**c**) Hematoxylin/eosin staining of liver sections. Hematopoietic centers present in liver are visible as groups of cells with intensely stained cell nuclei. Scale bars correspond to 50 µm in the main images, and to 20 µm in the magnified inserts. dpp – days postpartum.

In adult mice, heme and hemoglobin released from circulating erythrocytes during hemolysis are scavenged and metabolized by macrophages, including Browicz-Kupffer cells in the liver^7^. The *Hmox1* gene encodes HO1, an enzyme that catalyzes the rate-limiting step in the heme degradation pathway, which results in the formation of iron, CO and biliverdin^6^. *Hmox1* expression is mainly regulated at the transcriptional level^20^. We showed that the level of HO1 mRNA gradually increases in mice from day 3 to day 11 after birth (Fig. 2a), and displayed a opposite tendency to decreasing intensity of hemolysis as determined by the serum hemopexin level (Fig. 1b,c).

Although double immunofluorescence analysis of HO1 and the macrophage marker F4/80 in the liver clearly showed that HO1 is expressed in Browicz-Kupffer cells, this pattern of expression was only found in macrophages at the center of a ring of erythroblasts, so-called erythroblastic islands (Fig. 2b). These central macrophages are known to be specialized in providing nutrients to surrounding erythroblasts. Erythroblastic islands are widely accepted as morphological units involved in fetal erythropoiesis that are gradually lost from neonatal mouse livers^21^, as confirmed in Fig. 2b,c. The disappearance of erythroblastic islands is accompanied by degeneration and removal of the central macrophages, as can be clearly seen in liver sections from 11-day-old mice (Fig. 2b). Our findings provide a strong indication that liver macrophages are not primarily involved in the breakdown of heme derived from hemolyzed erythrocytes.

### Progressive disappearance of megalin-cubilin protein complex from renal proximal tubules during the neonatal period

**Figure 3:**
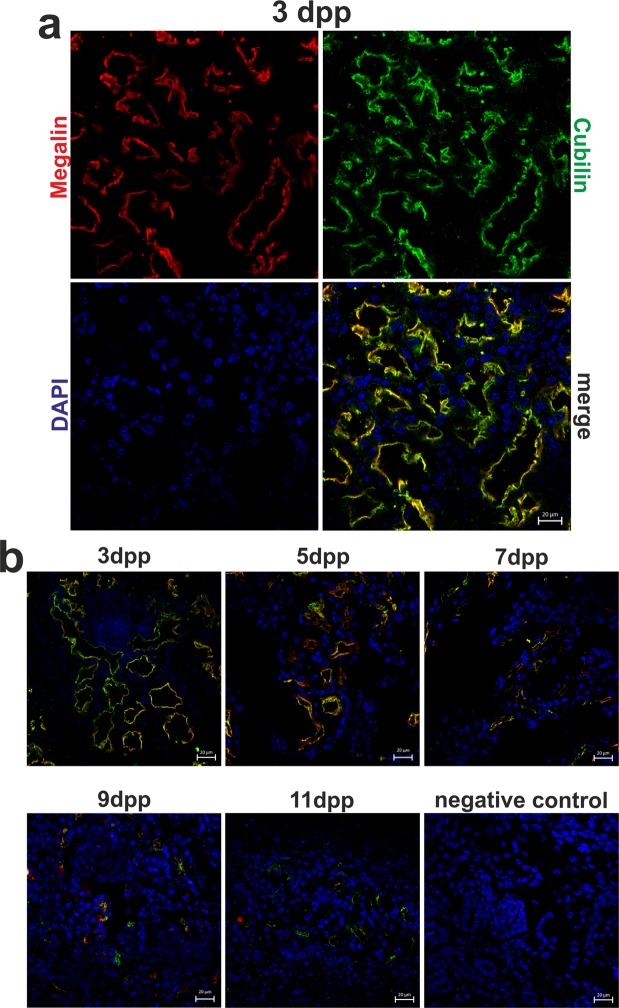
Immunofluorescent (IF) localization of megalin-cubilin complex in the renal proximal tubules of mice during the neonatal period. (**a**) IF staining of megalin (red channel) and cubilin (green channel) in the kidney of the 3-day-old mouse, analyzed by confocal microscopy, reveals that both transporters have apical localization in the epithelial cells of cortical renal tubules. Cell nuclei were counterstained with DAPI (blue). Scale bars correspond to 20 µm. (**b**) IF staining of megalin-cubilin complex in the kidneys of the 3-, 5-, 7-, 9- and 11-day old neonatal mice. analyzed by confocal microscopy, reveals that both transporters have apical localization in the epithelial cells of cortical renal tubules. Cell nuclei were counterstained with DAPI (blue). To confirm the specificity of megalin and cubilin detection, kidney sections were incubated only with the secondary antibodies (negative control). Scale bars correspond to 20 µm.

In the kidney, cubilin and megalin are involved in hemoglobin reabsorption in the renal proximal tubules^22^. Using the immunofluorescence (IF) method we found that megalin and cubilin are expressed in the renal cortical tubules of mouse neonates from all age groups (Fig. 3a,b). More precisely we showed that both megalin and cubilin are present in epithelial cells of the renal cortex and are localized in the apical region (Fig. 3a). We observed a relatively strong staining of megalin-cubilin complex in cortical renal tubules in mice on days 3, 5 and 7 postpartum. In 9- and 11-day-old mice the immunopositive IF signal was much weaker. (Fig. 3b). High expression of megalin-cubilin complex in the apical membrane of the proximal tubules of mouse neonates during first week of life strongly suggests its involvement in cellular reabsorption of hemoglobin during neonatal hemolysis.

### Progressive disappearance of HRG1 protein from renal proximal tubules during the neonatal period

**Figure 4:**
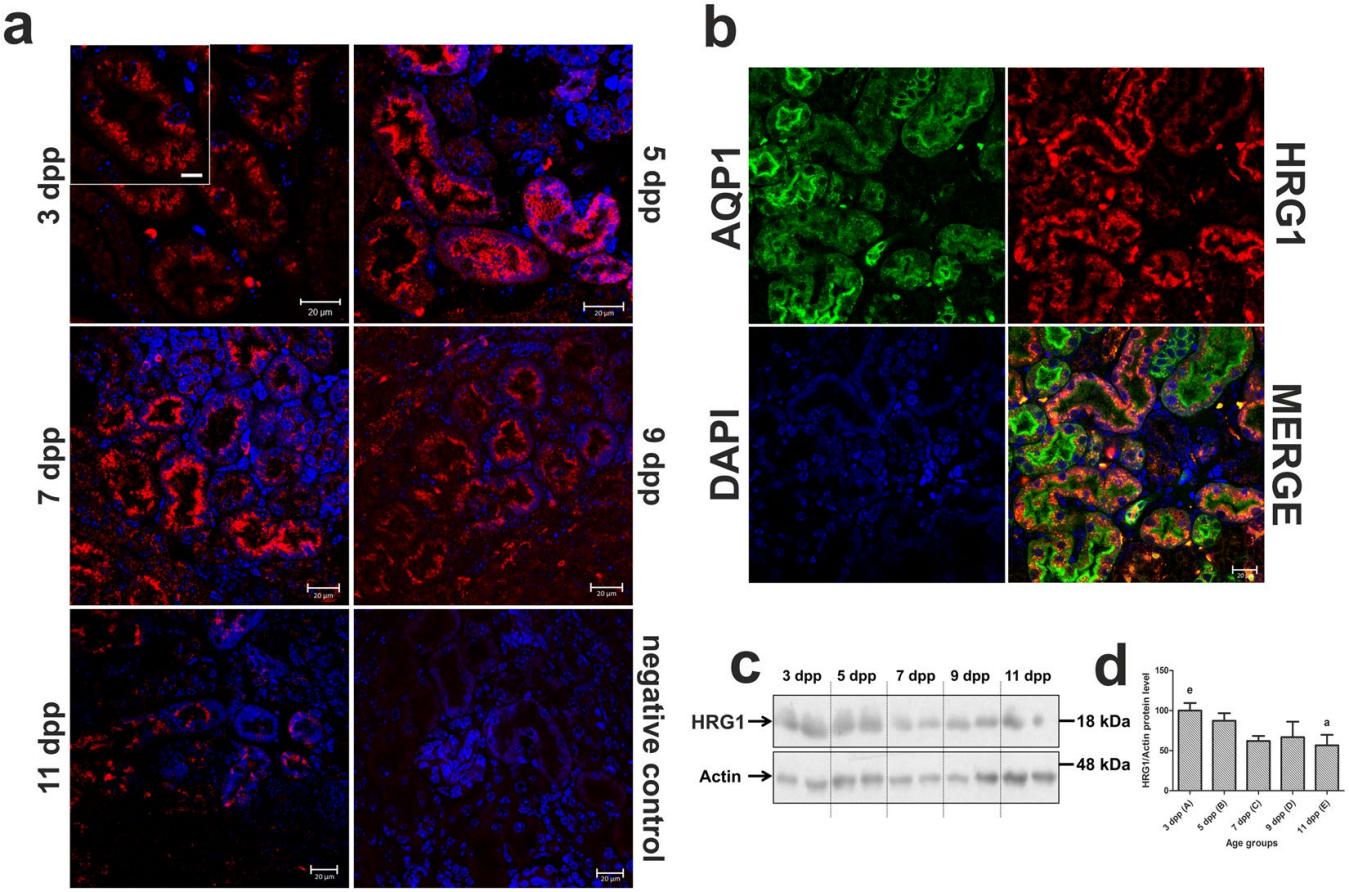
Immunofluorescent (IF) localization and quantitative analysis of heme-responsive gene 1 (HRG1/Slc48a1), a heme transporter, in the renal proximal tubules of mice during the neonatal period. (**a**) IF staining of HRG1/Slc48a1 (red channel) in the kidneys of mouse neonates, analyzed by confocal microscopy, reveals its apical and sub-apical localization in the epithelial cells of cortical renal tubules. Cell nuclei were counterstained with DAPI (blue). Scale bars correspond to 20 µm in the main images, and to 10 µm in the magnified insert. To confirm the specificity of HRG1/Slc48a1 detection, kidney sections were incubated only with the secondary antibody (negative control). (**b**) IF co-localization of HRG1/Slc48a1 (red channel) and aquaporin 1 (AQP1), a proximal tubule marker (green channel) in the kidneys of a 3-day old mouse. Cell nuclei were counterstained with DAPI (blue). Scale bars correspond to 20 µm. (**c**) Renal levels of HRG1/Slc48a1 assessed by Western blotting. The blot was reprobed with monoclonal anti-actin antibody as a loading control. (**d**) Immunolabeled HRG1/Slc48a1 and actin control bands were quantified using a Molecular Imager and the relative levels of HRG1/Slc48a1 (means ± S.D.) are plotted in arbitrary units (a.u). Results come from 2 separate blots and relative levels of proteins were standardized to a percentage with %S.D. in order to eliminate technical differences between blots. N values for each group are: 3dpp = 5, 5dpp = 5, 7dpp = 5, 9dpp = 5, 11dpp = 4. Data set for HRG1/Slc48a1 has normal distribution, therefore, one-way ANOVA was used (p = 0,0098, df = 4, F = 4,521). Tukey’s Multiple Comparison Test was used as post-hoc test. Small letters over the bars in the chart denote significant differences between age groups, with p < 0.05. dpp – days postpartum.

HRG1, the mammalian homolog of the *C. elegans* heme transporter^23^, has been identified as a protein that transfers heme across the phagosomal membrane to the cytosol within erythrophagocytosing macrophages^24^. HRG1 has also been detected on the apical membrane of duodenal enterocytes in piglets fed hemoglobin^25^. To assess the potential involvement of HRG1 in the transport of heme from the primary urine under conditions of neonatal hemolysis, we examined its localization and expression in the kidney. Immunofluorescence analysis revealed that HRG1 is present in epithelial cells of the renal cortex and is localized in apical/sub-apical regions (Fig. 4a). To determine the precise location of HRG1 in the nephron, we performed double immunolocalization with aquaporin 1 (AQP1), a specific marker for proximal tubules^26^. Tight co-localization of HRG1 and AQP1 was identified, which indicates that during the neonatal period, HRG1 is expressed in the proximal renal tubules (Fig. 4b). Time-course immunofluorescence (Fig. 4a) and Western blot (Fig. 4c,d) analyses of HRG1 levels between days 3–11 after birth clearly showed the progressive loss of this protein, suggesting its decreasing role in the transport of free heme from primary urine as hemolysis is reduced. Both localization of HRG1 on the epithelium of proximal tubules and its age-dependent expression pattern strongly suggest the active participation of this heme transporter in the clearance of heme from the primary urine during neonatal hemolysis in mice.

### Progressive disappearance of TfR1 and DMT1 proteins from neonatal renal proximal tubules

**Figure 5:**
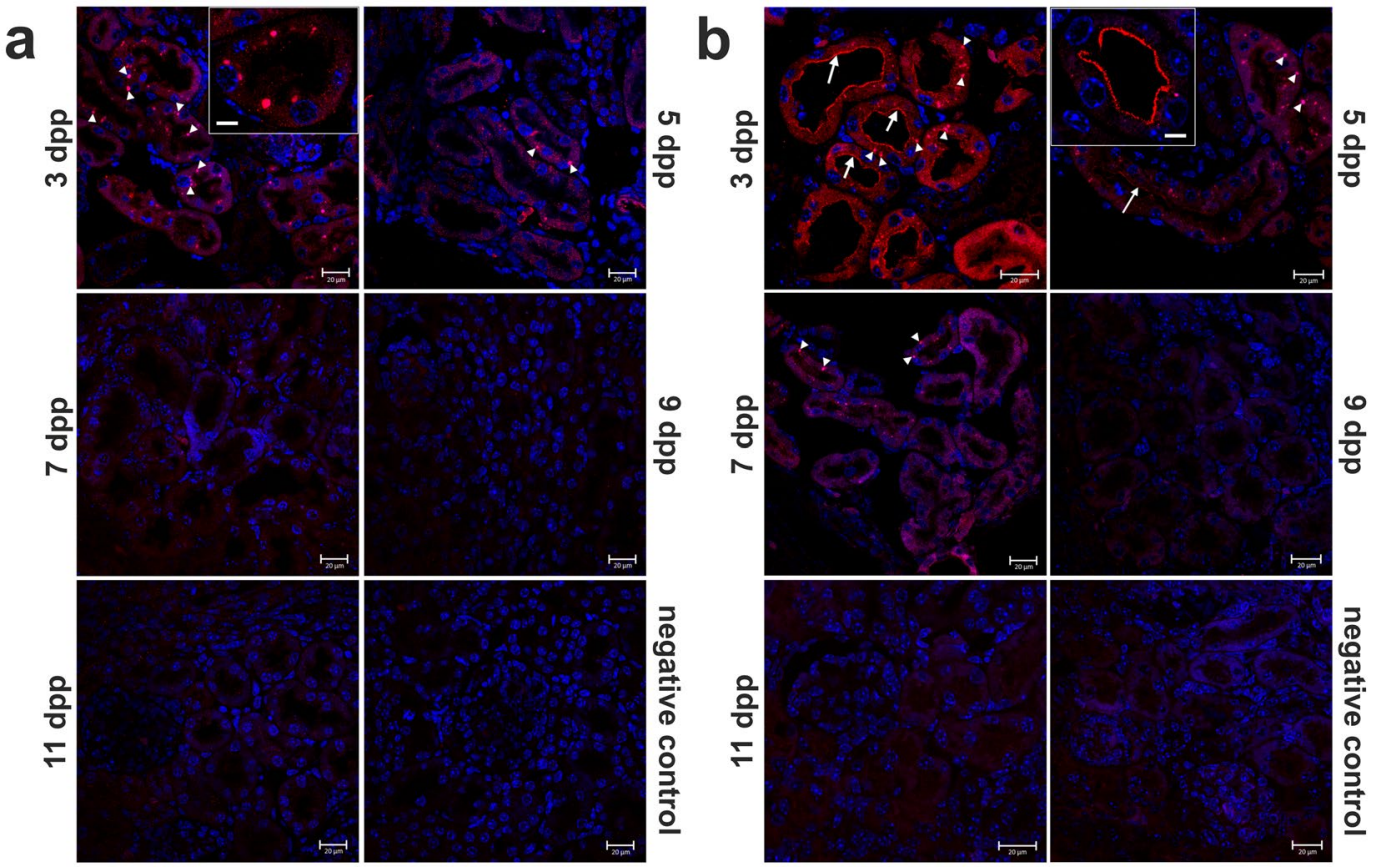
Immunofluorescent (IF) localization of transferrin receptor 1 (TfR1) and divalent metal transporter 1 (DMT1) proteins in the kidneys of mice during the neonatal period. (**a**) IF staining of TfR1 (red channel) in the kidneys of mouse neonates analyzed by confocal microscopy. Expression of TfR1 was detected in cytoplasmic vesicles located in the epithelial cells of cortical renal tubules (insert). A positive signal for TfR1 is visible only in 3- and 5-day-old mice. (**b**) IF staining of DMT1 (red channel) in the kidneys of mouse neonates analyzed by confocal microscopy. Expression was detected at the apical membrane of epithelial cells of the cortical renal tubules (insert and arrows) and in vesicles present in the cytoplasmic compartment (arrowheads). Apical localization of DMT1 was found only in kidneys of 3- and 5-day-old mice, whereas vesicular localization was detected in kidneys of 3-, 5- and 7-day old-mice. Cell nuclei were counterstained with DAPI (blue). To confirm the specificity of TfR1 and DMT1 detection, kidney sections were incubated with only the secondary antibody (negative control). Scale bars correspond to 20 µm in the main images, and to 10 µm in the magnified inserts.

During hemolysis, disrupted erythrocytes are a source of both non-heme and heme iron that leaks into the blood. Free iron ions are then rapidly bound by apo-transferrin and in complex with this protein they may be filtered out by glomeruli^27^, which show increased permeability in the neonatal period^28^. Diferric transferrin then binds to the high-affinity Tf receptor 1 (TfR1), present at the apical membrane of epithelial cells in the proximal tubules, and is reabsorbed from primary urine *via* TfR1-mediated endocytosis^29^. Transport of iron from the endosome to the cell cytosol is mediated by DMT1^30^. We analyzed renal expression and localization of TfR1 and DMT1 in mice throughout the neonatal period from day 3 to day 11 after birth. The greatest abundance of these proteins was found on the apical membrane of epithelial cells of cortical renal tubules in 3-day-old mice (Fig. 5a,b). Expression was then progressively reduced so that both proteins became almost undetectable in 9–11-day-old animals. We also observed DMT1 in intracellular vesicles of epithelial cells (Fig. 5b). This pattern of localization suggests that, according to a well-characterized mechanism, DMT1 participates in the transport of iron freed from transferrin within the endosome to the cytoplasm. Collectively, our results support the role of elemental iron importers in the uptake of non-heme iron by epithelial cells of proximal tubules from the primary urine during neonatal hemolysis in mice.

### Decrease in *Hmox1* gene expression and HO1 protein in neonatal renal proximal tubules

**Figure 6:**
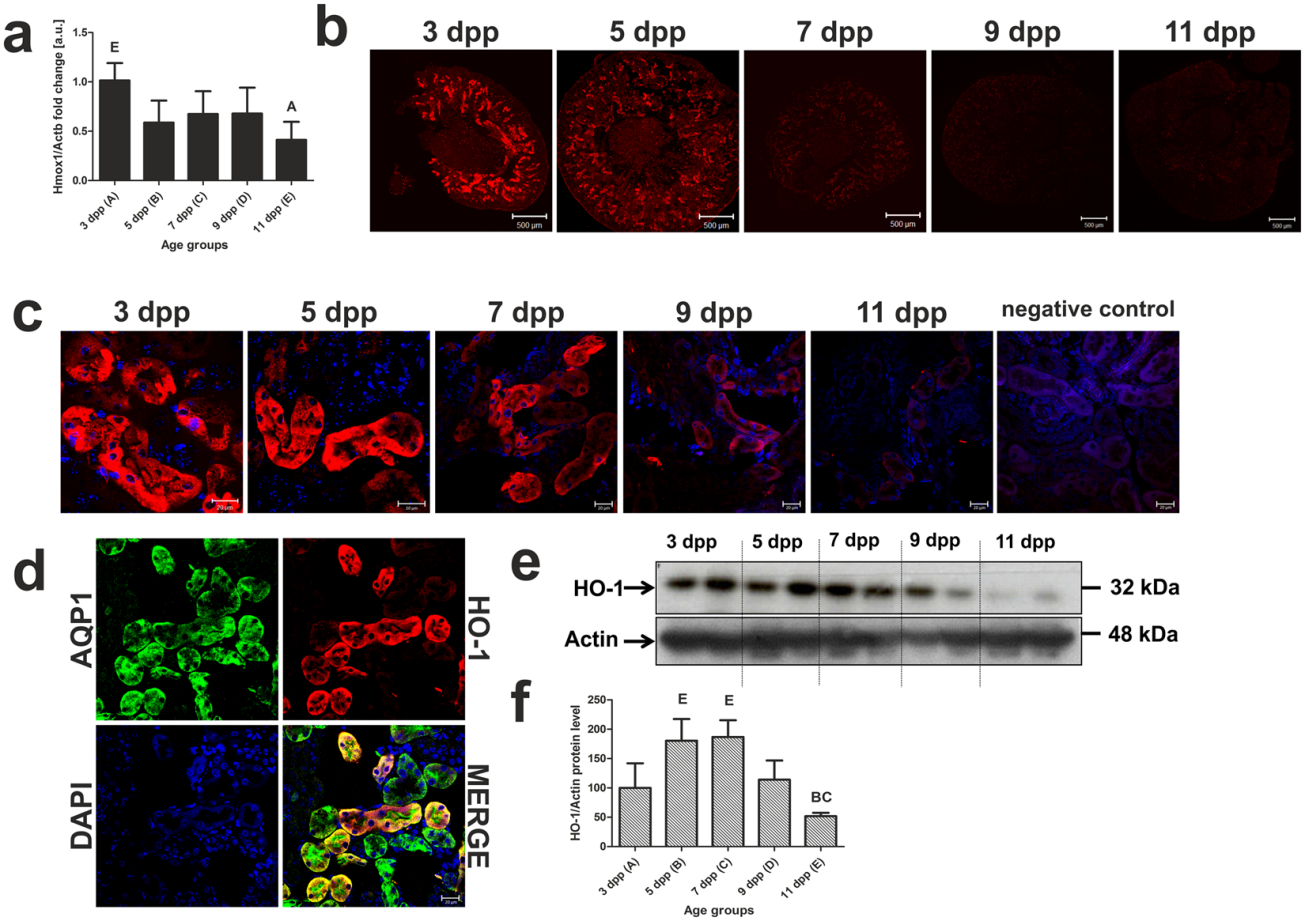
Immunofluorescent (IF) localization and quantitative analysis of heme oxygenase 1 (HO1) in the renal proximal tubules of mice during the neonatal period. (**a**) RT-qPCR analysis of HO1 transcript levels in the kidneys of mouse neonates from 3 to 11 days old. The histogram displays relative HO1 mRNA levels in arbitrary units (means ± S.D.). N values for each group are: 3dpp = 5, 5dpp = 4, 7dpp = 4, 9dpp = 4, 11dpp = 4. Data set for *Hmox1* expression has normal distribution, therefore, one-way ANOVA was used (p = 0,0101, df = 4, F = 4,569). Tukey’s Multiple Comparison Test was used as post-hoc test. (**b**) IF staining of HO1 in transverse sections of kidneys from mouse neonates. HO1 is exclusively expressed in the renal cortex. Scale bars correspond to 500 µm. (**c**) IF staining of HO1 (red channel) shows cytoplasmic protein localization in epithelial cells of the cortical renal tubules. Cell nuclei were counterstained with DAPI (blue). To confirm the specificity of HO1 detection, kidney sections were incubated with only the secondary antibody (negative control). Scale bars correspond to 20 µm. (**d**) IF co-localization of HO1 (red channel) and AQP1, a proximal tubule marker (green channel) in the kidney of a 3-day-old mouse. Cell nuclei were counterstained with DAPI (blue). The scale bar corresponds to 20 µm. (**e**) Levels of HO1 assessed in renal membrane extracts by Western blotting. The blot was reprobed with monoclonal anti-actin antibody as a loading control. (**f**) Immunolabeled HO1 and actin control bands were quantified using a Molecular Imager, and the relative levels of HO1 (means ± SD) are plotted in arbitrary units (a.u). Results come from 2 separate blots and relative levels of proteins were standardized to a percentage with %S.D. in order to eliminate technical differences between blots. N values for each group are: 3dpp = 5, 5dpp = 5, 7dpp = 5, 9dpp = 5, 11dpp = 4. Data set for HO1 has normal distribution, therefore, one-way ANOVA was used (p = 0,0039, df = 4, F = 5,564). Tukey’s Multiple Comparison Test was used as post-hoc test. Capital letters over the bars in charts (**a**) and (**f**) denote significant differences between age groups, with p < 0.01. dpp – days postpartum.

Hemolytic disorders are known to induce *Hmox1* expression in the kidney in both adult humans^9,10^ and laboratory animals^11^. Therefore, we examined whether under conditions of moderate hemolysis, the production of HO1 is up-regulated in the kidney of mouse neonates, especially since we demonstrated the increased molecular renal potential to take up heme (a strong inducer of *Hmox1*) from primary urine (Fig. 4). RT-qPCR analysis showed that an initially high level of the HO1 transcript on day 3 after birth was reduced by about half on successive days to attain a significantly lower level on day 11 (Fig. 6a) and displayed a similar tendency as decreasing intensity of hemolysis. Immunofluorescence analysis of transverse kidney sections showed a strong HO1 protein signal in cortical renal tubules on days 3 and 5 postpartum, but this had almost disappeared in 9- and 11-day-old mice (Fig. 6b). Exactly the same pattern of HO1 expression was seen in the epithelial cells of renal tubules examined at high magnification (Fig. 6c). Co-localization analysis revealed an overlap in the staining of HO1 and AQP1, indicating that similarly to HRG1, HO1 is expressed in the epithelial cells of the proximal renal tubules. The pattern of HO1 expression was corroborated by Western blot analysis of renal membrane extracts, which showed a progressive decrease in HO1 protein abundance between days 3–11 (Fig. 6e,f). In the view of these results, HO1 located in the epithelial cells of proximal tubules is mainly responsible for the breakdown of heme taken up from the primary urine during neonatal hemolysis in mice.

### Iron status in the neonatal kidneys

**Figure 7:**
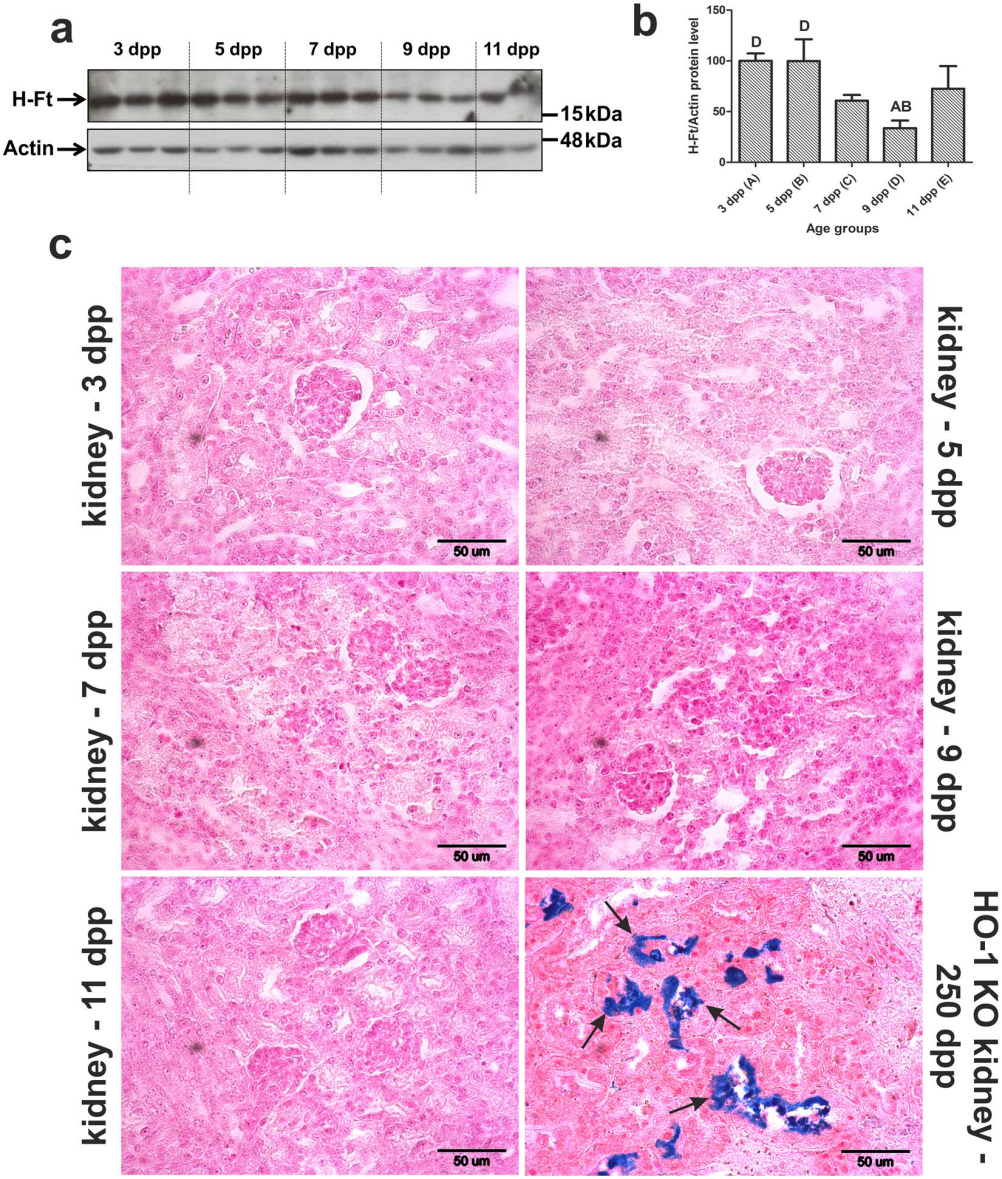
Changes in H-ferritin (H-Ft) protein level and the absence of iron deposits in the kidneys of mice during the neonatal period. (**a**) Levels of H-Ft assessed in renal cytosolic extracts by Western blotting. The blot was reprobed with monoclonal anti-actin antibody as a loading control. (**b**) Immunolabeled H-Ft and actin control bands were quantified using a Molecular Imager and the relative levels of H-Ft (means ± S.D.) are plotted in arbitrary units (a.u). Results come from 2 separate blots and relative levels of proteins were standardized to a percentage with %S.D. in order to eliminate technical differences between blots. N values for each group are: 3dpp = 5, 5dpp = 5, 7dpp = 5, 9dpp = 5, 11dpp = 4. Data set for H-Ft has normal distribution, therefore, one-way ANOVA was used (p = 0,0014, df = 4, F = 6,849). Tukey’s Multiple Comparison Test was used as post-hoc test. Capital letters over the bars in the chart denote significant differences between age groups, with p < 0.01. (**c**) Histological examination of iron loading in the kidneys of mouse neonates. No non-heme iron deposits were detected by staining with Perls’ Prussian Blue. Kidney section from 250-day-old HO1 knock-out mouse was used as a positive control of iron loading in renal cortex (indicated by arrows) during excessive hemolysis^37^. dpp – days postpartum.

HO1-mediated heme breakdown results in the release of ferrous iron, which is a strong inducer of ferritin expression^31^. Ferritin is a cytosolic protein with a high capacity to store potentially toxic iron, and thus plays a vital cytoprotective role^32^. We used Western blotting to analyze the renal level of the H-ferritin subunit (H-Ft) possessing ferrooxidase activity, which is crucial for the incorporation of iron into the ferritin molecule^33^. Determination of H-Ft protein levels in the kidneys of 3–9-day-old mice, revealed a pattern of decreasing expression similar to HO1, suggesting tight cooperation between these two proteins in detoxifying both heme and non-heme iron (Fig. 7a,b). A slight increase in the level of H-Ft in 11-day-old mice appeared to be related to developmental changes in the systemic iron metabolism of this species^34^. Iron complexed with ferritin can be readily detected by histochemical analysis, i.e. by staining tissue sections with Perls’ Prussian blue, which results in the appearance of blue deposits. We used this method to examine kidneys from mouse neonates but failed to detect iron deposits in kidney sections across the renal cortex (Fig. 7c). These results suggest that while H-Ft is induced in the kidney during neonatal hemolysis, it does not form iron deposits because the released iron is expelled from the cells *via* ferroportin.

### Hepcidin-independent progressive decrease in ferroportin level in renal proximal tubules during the neonatal period

**Figure 8:**
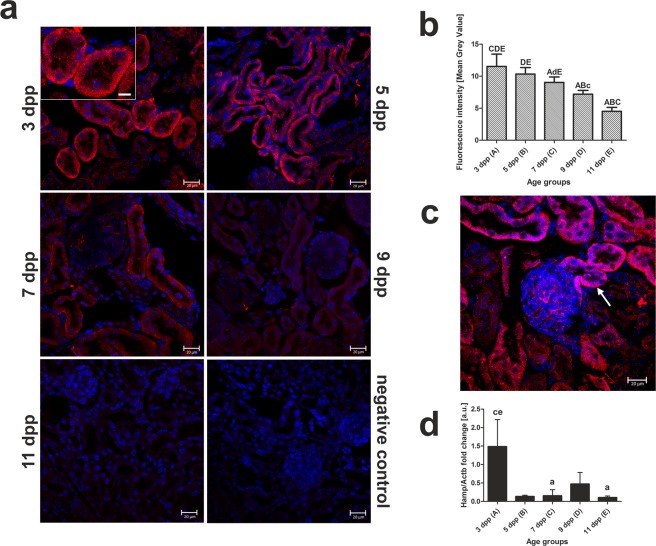
Immunofluorescent (IF) localization and quantitative analysis of ferroportin (Fpn) in the kidneys of neonatal mice combined with quantitative expression analysis of *Hamp* gene in livers of experimental mice. (**a**) IF staining of Fpn (red channel) in the epithelial cells of cortical renal tubules of mouse neonates, analyzed by confocal microscopy. A positive signal is visible on the basolateral membrane of renal cortex epithelial cells in 3- (magnified insert), 5- and 7-day-old mice. Cell nuclei were counterstained with DAPI (blue). To confirm the specificity of Fpn detection, kidney sections were incubated only with the secondary antibody (negative control). Scale bars correspond to 20 µm in the main images, and to 10 µm in the magnified insert. (**b**) Quantitative analysis of the Fpn fluorescent signal in renal cortical tubules. The mean fluorescence signal associated with Fpn in each tubule section was measured by ImageJ analysis (3 individuals per age group, 10 measurements per individual, N = 30 per each age group) and quantified manually as a Mean Grey Value, the intensities (means ± S.D.) are plotted in arbitrary units (a.u). Data set for Fpn is not normally distributed, therefore, nonparametric, Kruskal-Wallis ANOVA was used (p < 0.0001, df = 4, H = 125,4). Dunn’s Multiple Comparison Test was used as post-hoc test. (**c**) Basolateral Fpn localization in the initial segment of the nephron (arrow) reveals that this protein is present in the proximal renal tubules. Cell nuclei were counterstained with DAPI (blue). The scale bar corresponds to 20 µm. (**d**) RT-qPCR analysis of *Hamp* transcript levels in the livers of mouse neonates from 3 to 11 days old. The histogram displays relative *Hamp* mRNA levels in arbitrary units (means ± S.D.). N values for each group are: 3dpp = 4, 5dpp = 5, 7dpp = 4, 9dpp = 4, 11dpp = 4. Data set for *Hamp* expression is not normally distributed, therefore, nonparametric, Kruskal-Wallis ANOVA was used (p = 0,0053, df = 4, H = 14,74). Dunn’s Multiple Comparison Test was used as post-hoc test. Capital and small letters over bars in the charts (**b**,**d**) denote significant differences between age groups, with p < 0.01 and p < 0.05, respectively. dpp – days postpartum.

Ferroportin (Fpn), the sole iron exporter known to date, is mainly expressed in duodenal enterocytes and macrophages^35^. However, it has also been detected in proximal tubule cells in both rat^36^ and mouse kidney^37^. It has been proposed that by transporting iron across the basolateral membrane of tubular cells to the blood Fpn is involved in the traffic of iron reabsorbed from the primary urine^12^. We therefore examined the expression and localization of Fpn as a plausible final element in the transport of iron across renal proximal tubules during the neonatal hemolysis period. Immunofluorescent detection of Fpn in mouse neonate kidney sections (Fig. 8a) followed by quantitative analysis of the fluorescent signal, showed a decrease in abundance (with the highest and lowest levels on days 3 and 11, respectively) (Fig. 8b), which mirrored that observed for the other iron and heme transporters and HO1 (Figures 1–6). Magnification of IF-stained renal cross-sections through the first part of the nephron revealed that Fpn is expressed in the proximal tubules (Fig. 8c). To investigate the potential role of hepcidin, (liver-derived peptide that binds to Fpn, and induces its degradation^13^) in the regulation of Fpn protein level on the basolateral membrane of tubular cells we measured hepcidin mRNA abundance in the liver during the neonatal period. Our results indicate that gradual decrease in Fpn in the kidneys of mouse neonates is largely hepcidin-independent (Fig. 8d). Based on our results, Fpn is a candidate protein charged to remove excess iron from epithelial cells of proximal tubules into the bloodstream during neonatal hemolysis in mice.

## Discussion

Over the last 10 years, it has become apparent that the kidneys play an important role in systemic iron metabolism^12^. The renal tubular epithelial cells are thought to represent novel iron storage and recycling sites. Indeed, these cells are equipped with the molecular machinery necessary for the detoxification of heme and non-heme iron as well as the vectorial transport of this microelement across the proximal tubule epithelium^13,14,38^. Furthermore, the iron metabolism in the kidneys is regulated systemically^13^ and locally^39^ by hepcidin and intracellularly by iron regulatory proteins^29^. Numerous examples from human patients^9^ and experimental animals^10,11^ provide evidence of the involvement of the kidneys in handling iron entering the circulation as a result of intravascular hemolytic disorders. Under these conditions, hemoglobin (Hb), a highly reactive pro-oxidant and potentially toxic species, is released from ruptured erythrocytes into the blood plasma. In most mammals toxicity of free Hb is efficiently neutralized by a multicomponent molecular mechanism composed of haptoglobin (Hp), a high-affinity Hb-binding protein and CD163, a scavenger receptor of the Hb-Hp complex present on macrophages/monocytes^40^. However, when endogenous Hp is depleted by accelerated hemolytic conditions, free Hb traverses the glomerular filtration barrier and appears in the urinary space from where it is endocytosed and metabolized by the renal proximal tubules. Interestingly, CD163-mediated scavenging of Hb in mice is largely independent of Hp-Hb complex formation^17^.

In this study, taking into account both the increased RBC breakdown in neonates (the primary cause of bilirubinemia in this group)^2^ and the functional immaturity of neonatal liver to cope with a hemolytic insult^21^, we used mouse neonates aged between 3- and 11-days-old to investigate the impact of increased neonatal hemolysis on the expression of iron-handling proteins in the mouse kidney. Over the period covered by the study, the neonatal mice showed increased serum bilirubin, a useful marker of accelerated RBC damage^2^. To our knowledge, this is the first description of the day-by-day serum bilirubin concentration in newborn mice, thereby confirming the occurrence of physiological bilirubinemia in the neonatal period. The intensity of this bilirubinemia was much lower than that induced in genetically modified animals with impaired neonatal bilirubin conjugation^15^. RBC indices show gradually decreasing hemolysis of fetal erythrocytes and thus reflects developmental trends for newborn healthy mice described by others^18^. The extent of intravascular hemolysis can also be evaluated by the depletion of plasma hemopexin (Hx), a plasma glycoprotein able to bind heme with high affinity and transport it to the liver for catabolism^5,41^. We therefore additionally diagnosed hemolysis in newborn mice by measuring the plasma Hx level and showed its progressive increase during the neonatal period, indicating the gradual recovery of the animals from hemolysis. Gradual disappearance of hemolysis in mouse neonates between days 3 and 11 after birth was also confirmed by decreasing level of LDH2 in the serum^19^. In adult mice, liver is the main site of final Hb and heme detoxification in a process involving the catalytic activity of HO1, encoded by the *Hmox1* gene, which is transcriptionally upregulated by a number of stimuli including heme^6^. We found that the age-dependent rise in hepatic HO1 mRNA level displayed an opposite tendency to intensity of hemolysis in mouse neonates. This strongly suggests that the liver is not the primary site of HO1-mediated removal of toxic heme from the circulation. The HO1 protein in mouse neonatal liver seems to be mainly localized within macrophages in the so-called erythroblastic islands, composed of a central macrophage and surrounding erythroblasts, and widely accepted as the morphological units responsible for fetal erythropoiesis^21^. HO1 activity in these macrophages may be associated with the progressive degradation of these erythroblastic islands during the neonatal and early postnatal periods^21^.

The demonstration of the decreased capacity of the liver to detoxify hemoglobin and heme released during hemolysis in mouse neonates prompted us to investigate the role of the kidney in this function. Earlier plasma clearance studies in mice showed the rapid elimination of Hb from the circulation, with the highest uptake by the kidney and liver^17^. Hb, if not bound by Hp, readily passes through the glomerular filter to enter the primary urine, from where it is rapidly cleared by the renal proximal epithelium^5,41^ via the multi-ligand hetero-dimeric receptor-complex megalin/cubilin^42^. Renal uptake of hemoglobin by megalin/cubilin complex has been reported in hemolytic conditions^43^. Herein, we demonstrate for the first time that the expression of these two receptors in renal proximal tubules gradually decreases with age in mouse neonates, suggesting their involvement in cellular reabsorption of hemoglobin during neonatal hemolysis.

It is known that extracellular Hb undergoes oxidation to higher oxidation states such as metHb, which readily releases heme molecules^44^. By sequestering Hb-derived heme within a protein complex, Hx prevents its pro-oxidant and pro-inflammatory effects and promotes its detoxification. The crucial role of Hx in preventing severe renal damage following hemolytic processes has been clearly demonstrated in Hx knock-out mice^45^.

Substantial changes in renal iron metabolism following exposure to heme and non-heme iron caused by acute hemolytic disorders have been well documented in patients^9^ and experimental animals^10,11^. However, despite the induction of antioxidant cytoprotective proteins such as HO1 and ferritin (Ft), the kidneys remain sensitive targets of iron toxicity following exposure to hemoglobin and heme during hemolytic anemias^46,47^. Here, we showed that mild hemolysis in mouse neonates, which occurs without anemia, results in substantial remodeling of the renal iron metabolism, which is most intense in 3-day-old mice and then gradually disappears in older animals, concomitantly with the decline in hemolysis. We hypothesize that, in addition to loading of the kidney with heme iron complexed with Hb, free Hb-derived heme is directly taken up from the tubular lumen *via* a pathway involving HRG1. HRG1 was identified as the main importer of exogenous heme in *Caenorhabditis elegans*, a heme auxotrophic nematode^23^. In addition, HRG1 localized on the phagosomal membrane was found to transport heme from the phagolysosome to the cytosol of mouse macrophages during erythrophagocytosis^48^. The role of HRG1 in heme-iron recycling has recently been further demonstrated during hemolysis in the zebrafish kidney, the major organ for erythrophagocytosis^49^. In the present study, we have demonstrated for the first time that HRG1 is expressed on the apical membrane of proximal tubular epithelial cells in mouse kidney. The level of HRG1 expression at this location decreased with age in mouse neonates and was tightly correlated with the retreat of hemolysis, suggesting a role for HRG1 in the transport of heme from the proximal tubular lumen to the proximal epithelium.

In recent years, it has become clear that transferrin (Tf)-bound iron may be filtered by the glomerulus into the tubular lumen^27^ and may subsequently be completely reabsorbed from the primary urine by TfR1-mediated endocytosis^29^. Successive transport of iron from the endosome to the cell cytosol is mediated by DMT1^29^. We showed that both proteins involved in the transport of non-heme iron are highly expressed in the epithelial tubular cells of 3–5-day-old mice. This indicates that under conditions of accelerated defense in cells and tissues, including the kidneys, loaded with heme iron^50^. Increases in both the HO1 mRNA and protein in renal tissue has been reported in various hemolytic conditions^9–11^. Our comprehensive analysis of HO1 expression and localization in the kidneys of mouse neonates demonstrated a marked induction of both the transcript and protein in 3-day-old mice. HO1 expression occurred at defined locations throughout the renal cortex. More precisely, the epithelial cells of proximal tubules were identified as the principal cells expressing HO1, which corresponds to the typical renal localization observed in hemolytic syndromes^9–11,50^. The time-course of the disappearance of renal HO1 expression in mouse neonates displayed a similar tendency as decreasing intensity of hemolysis, which supports a protective role for this enzyme during neonatal hemolysis. As originally demonstrated by Balla *et al*.^32^, in mouse endothelium HO1 acts in concert with ferritin for the efficient detoxification of both heme and non-heme iron. HO1-mediated heme breakdown is followed by the induction of ferritin, which binds and neutralizes potentially toxic heme-derived iron. The cooperation between these proteins has been well established in cellular and *in vivo* models^31^. Systemic knockout in mice of either the *Hmox1* gene^37^ or a proximal tubule-specific knock-out of the *H-Ft* gene^51^ (encoding the ferritin heavy chain)^33^ resulted in severe injury of the kidneys. The age-dependent renal expression pattern of H-Ft protein in mouse neonates strongly resembles that of HO1, which underscores the protective HO1/H-Ft partnership that is active during neonatal hemolysis. Iron-regulated induction of ferritin expression in hemolytic disorders is usually associated with an increased amount of iron stored in a non-toxic form within ferritin molecules. However, in contrast to a congenital erythropoietic porphyria, a chronic mouse model of hemolytic disorder^43^, we failed to detect any rise in iron content in the kidneys of mouse neonates by staining renal sections with Prussian blue, even during the period of highest hemolysis (days 3–7 after birth). Although this lack of staining does not exclude limited iron accumulation in ferritin (which is below the detection limit of the method), it certainly excludes the occurrence of heavy iron deposits observed in the kidneys during pathological hemolytic conditions^9–11,50^. The absence of this phenomenon may reflect the moderate nature of neonatal hemolysis in newborn mice and also the very high up-regulation of ferroportin (the main iron export protein), leading to increased efflux of iron from the proximal tubule epithelium into the circulation. Indeed, we found that ferroportin is mainly localized on the basolateral membrane of proximal epithelial tubule cells, which supports its function as an iron exporter. This basolateral localization of ferroportin was previously described in both rat^36^ and mouse^37^ kidney. It is noteworthy that the neonatal pattern of Fpn expression in the renal proximal tubule epithelium matches the expression pattern of other iron- and heme-related proteins analyzed in this study. Increase in protein abundance of Fpn, HO1 and ferritin in the cortical part of the kidney was reported in adult mice with severe hemolytic disorder such as congenital erythropoietic porphyria^43^. Interestingly, it appears that renal Fpn expression in mouse neonates is not systematically regulated by hepcidin, similarly to previous reports^52,53^, as hepatic expression of the *Hamp* gene was induced only on day 3 postpartum and during consecutive days of neonatal period was barely detectable. Hepcidin expression was also reduced in the hemolytic disease mentioned above and in this particular pathology down-regulation of hepcidin was associated with the erythropoiesis-induced expression of *Fam132b* gene encoding erythroferrone, a known suppressor of hepcidin synthesis^54^. The pattern of decreased Fpn expression in the kidney of mouse neonates strongly suggests that it is mainly regulated by weakening intracellular signal strength from non-heme and heme iron^55^.

Taken together, the findings of this study provide evidence that the proximal epithelial tubule cells of mouse neonates are equipped with a powerful molecular machinery for the efficient recovery of heme and non-heme iron from the primary urine, its intracellular detoxification, and the redistribution of non-heme iron to the blood plasma. During the period of neonatal hemolysis, in a situation of temporary physiological liver failure, the kidneys play a vital role in iron detoxification and recycling in the body. The latter function is crucial for preventing the loss of iron from the organism. The part played by the kidneys in favoring iron retention in the body of neonates helps to balance the demand for this microelement for erythropoiesis and other iron-dependent processes. This is particularly important given that the supply of exogenous iron is inadequate in the neonatal period due to the well-known functional immaturity of the molecular mechanisms of iron absorption^56^.

## Materials and Methods

### Animals

The mice derived from an outbred colony, bred in the Department of Genetics and Evolution, Jagiellonian University. We examined 3-, 5-, 7-, 9- and 11-day-old male mice, with the day of birth counted as day 1. Up to the day of sacrifice all pups stayed with their mothers. Some experiments were performed on adult, 90-day-old males. All mice were housed at constant temperature (22 °C) under artificial light (12-hour photoperiod). The mothers and adult mice received a standard diet (Labofeed H, Kcynia, Poland) and water *ad libitum*. Neonatal mice were sacrificed by decapitation, while adult mice were sacrificed by isoflurane (Baxter) overdose. All animal procedures were in accordance with *Guide for the Care and Use of Laboratory Animals* (Directive 2010/63/EU of European Parliament). However, as the only procedure used in this work was post mortem tissue collection we have got an approval of the First Local Ethical Committee on Animal Testing at the Jagiellonian University in Krakow (permission number: 136/2015).

### Sample collection

Blood samples were taken from neonates using heparinized tips or from the inferior vena cava of adult males using heparin-coated syringes. The blood was collected in heparin-coated tubes and centrifuged at 800 g/4 °C/10 min. Plasma samples were then frozen in liquid nitrogen and stored at −80 °C for further analysis. Renal and hepatic samples were frozen immediately after the dissection. Tissue samples for immunofluorescence analysis were fixed in 4% paraformaldehyde in PBS. For the preparation of paraffin-embedded sections, livers were fixed in Bouin’s solution for 24 h.

### Measurement of plasma bilirubin concentration

Samples of frozen blood plasma from neonatal and adult males were thawed on ice and centrifuged at 800 g/4 °C/10 min. Total bilirubin (T-Bil) concentrations were measured using SPOTCHEM Total Bilirubin reagent strips with a SPOTCHEM EZ SP-4430 biochemical analyser (Arkray) according to the manufacturer’s instructions.

### Hematological parameters measurements

Blood samples were collected to heparin-coated tubes and kept on ice. Red blood cells count (RBC), mean corpuscular volume of a red blood cell (MCV) and red cell distribution width (RDW) were measured using a hematology analyser (ABC Vet Animal Blood Counter, Horiba ABX) according to the manufacturer’s instructions.

### Western blot analysis

To determine plasma haptoglobin (Hp) and hemopexin (Hpx) levels, 5 µl samples of 20-fold diluted mouse blood plasma were resolved by electrophoresis on 8% SDS-PAGE gels. To analsyse LDH2 levels, 7 µl samples of 25-fold diluted mouse blood plasma were resolved by electrophoresis on 10% SDS-PAGE gels. Heme oxygenase 1 (HO1) and Slc48a1/HRG1 proteins were detected in 40 µg of membrane extracts (prepared as described previously^37^) following electrophoresis on non-denaturing 10% SDS-PAGE gels. Electroblotting of the resolved proteins onto PVDF membrane (Millipore), blocking and incubation with primary antibodies were performed as described previously^37^. The primary and secondary antibodies used for immunoblotting experiments are described in Table S1. For quantitative analysis of protein content, reactive bands were quantified relative to those of albumin or actin using a Molecular Imager with Quantity One software (Bio-Rad).

### Real-time quantitative RT-PCR

Total RNA was isolated from kidneys and livers of experimental animals using an RNA isolation kit (Macherey-Nagel). The RNA was treated with DNAse-I (Macherey-Nagel) and reverse transcribed using a High-Capacity cDNA Reverse Transcription Kit (Applied Biosystems). A specific *Hmox1* and *Hamp* fragments were then PCR amplified from this cDNA using the specific primers (Table S2). To evaluate the relative expression level compared to *Actb*, the deltadelta cT method was used. Real time qPCR was performed with the SybrGreen quantitative PCR kit (Applied Biosystems) using a StepOne thermocycler (Applied Biosystems).

### Immunofluorescence (IF) analysis and confocal microscopy of liver and kidney sections

After laparotomy, mouse livers and kidneys were immediately dissected and fixed in 4% paraformaldehyde (Sigma) in phosphate-buffered saline (PBS) at 4 °C for 24 h. Following two 30 min washes in PBS, the tissues were successively soaked in 12.5 and 25% sucrose (Bioshop) for 2 h and 7 days, respectively, at 4 °C. Hepatic and renal samples were then embedded in Cryomatrix medium (Thermo Scientific), frozen in liquid nitrogen and sectioned in 20-μm slices using a cryomicrotome (Shandon). The sections were washed in PBS for 10 min and permeabilized by bathing in PBS/0.1% Triton X-100 (Sigma) for 20 min. Non-specific antibody binding was blocked by incubating the tissue sections in PBS/3% BSA (Bioshop) for 1.5 h. For protein detection, sections were incubated overnight at room temperature with primary antibodies diluted in PBS/3% BSA. As a negative control, some sections were incubated without primary antibody. The primary and fluorochrome-conjugated secondary antibodies used in IF analysis are described in Supplementary Table S3. Next, the sections were washed for 5 × 6 min with PBS/0.1% Triton X-100 and incubated for 1.5 h with secondary antibody diluted in PBS/3% BSA at RT. Finally, sections were washed for 10 min in PBS and mounted in Vectashield medium with DAPI (Vector Labs). The presence of HO1 and Fpn in Browicz-Kupffer cells (liver macrophages) was determined by double immunofluorescence localization of the investigated proteins and the macrophage marker F4/80. In order to distinguish the different proteins in this experiment, the secondary antibodies were conjugated with different fluorochromes: Cy3 for HO1/Fpn and Alexa488 for marker F4/80. For the immunolocalization of HO1 and F4/80, the standard IF protocol was performed using mixtures of the required primary and secondary antibodies. For the immunolocalization of Fpn and F4/80, liver sections were first incubated with anti-Fpn and then with anti-F4/80 primary antibodies, then they were incubated with the mixture of secondary antibodies. The presence of HO1 and HRG1 in kidney was determined by double immunofluorescence localization of the investigated proteins and the marker AQP1, using secondary antibodies conjugated with different fluorochromes. Because the primary antibodies for the investigated proteins and for marker AQP1 were produced in the same host (rabbit), a protocol for fluorochrome-conjugated AffiniPure Fab fragments (Jackson Immunoresearch) was used for blocking and labelling primary antibodies. Monovalent Fab fragments of affinity-purified secondary antibodies were used to block the surface of specific primary antibodies. Because each labelled Fab fragment has only a single antigen binding site this prevents cross-reaction between the different secondary antibodies. HO1 and HRG1 were bound with primary antibodies, and these were then both blocked and labelled with Cy3-conjugated Fab fragments. Subsequently, the marker protein AQP1 was localized with primary antibodies and labelled with a standard divalent Alexa488-conjugated secondary antibody. IF was analyzed with a Zeiss LSM 710 confocal microscope (Carl Zeiss) using a ×40 objective and Zeiss ZEN software.

### Histological analysis of liver sections

Following fixation in Bouin’s solution for 24 h, livers were stored in 70% ethanol before further preparation. The tissue was embedded in paraffin and 7-μm cross-sections were cut through liver lobes with a microtome (Reichert-Jung, Germany). The sections were stained with hematoxylin and eosin. Slides were examined by light microscopy (Olympus, type CH2).

### Renal iron staining

Non-heme iron deposits were analyzed using an Accustain Iron Deposition Kit (Sigma Aldrich). Briefly, kidney samples were excised immediately after sacrifice, fixed in Bouin’s solution for 24 h, then stored in 70% ethanol. After embedding in paraffin, the samples were cut into 7-μm sections using a microtome (Reichert-Jung, Germany). The sections were placed on a slide, deparaffinised and hydrated tissues were incubated with a working solution containing Perls’ Prussian Blue for 30 min. Then counterstained with pararosaniline solution for 2 min and analyzed by standard light microscopy (Olympus, type CH2).

### ImageJ analysis of immunofluorescence images

ImageJ analysis was performed to characterize immunofluorescence produced using the ferroportin antibody. Samples from each group of animals were prepared for analysis at the same time. Kidney sections from 3 different individuals per age group were examined by confocal microscopy at 40× magnification. For quantitative comparisons between age groups, the same acquisition parameters were applied to images within the same experimental set. Subsequently, ImageJ software (NIH, Bethesda) was used to measure mean fluorescence in each tubule section. The signal intensity was manually quantified to generate a Mean Gray Value: the sum of grey values in the selected area divided by the number of pixels within that area. For each experimental group, 30 measurements of cortical renal tubules were made (10 measurements per individual).

### Statistical analysis

Data were analyzed for normal distribution using Shapiro–Wilk test. Differences between groups were compared by parametric, two-tailed ANOVA tests or non-parametric, two-tailed Kruskal-Wallis ANOVA tests combined with proper post-hoc tests (Tukey test and Dunn test, respectively). A value of p < 0.05 being considered as statistically significant.

## Supplementary information

Supplementary Materials

## Data Availability

The datasets generated and analyzed during the current study are available from the corresponding author on reasonable request.
