# Supplementary material for: Role of the kidneys in the redistribution of heme-derived iron during neonatal hemolysis in mice
Source: Sci Rep. 2019 Jul 31;9:11102. doi: 10.1038/s41598-019-47414-y (PMC6668426; doi:10.1038/s41598-019-47414-y)

**Role of the kidneys in the redistribution of heme-derived iron during neonatal hemolysis in mice**

Aleksandra Bednarz^1^ MSc, Paweł Lipiński^2*^ Prof., Rafał R. Starzyński^2^ PhD, Mateusz Tomczyk^3^ MSc, Witold Nowak^3^ PhD, Olga Mucha^3^ MSc, Mateusz Ogórek^1^ MSc, Olga Pierzchała^1^ MSc, Aneta Jończy^2^ MSc, Robert Staroń^2^ PhD, Julia Śmierzchalska^1^ MSc, Zenon Rajfur^4^ PhD, Zbigniew Baster^4^ MSc Eng, Alicja Józkowicz^3^ Prof., Małgorzata Lenartowicz^1*^ PhD

^1^Department of Genetics and Evolution, Institute of Zoology and Biomedical Research, Jagiellonian University, Gronostajowa 9, 30-387 Kraków, Poland

^2^Department of Molecular Biology, Institute of Genetics and Animal Breeding, Polish Academy of Sciences, 05-552, Magdalenka, Jastrzębiec, Poland

^3^Department of Medical Biotechnology, Faculty of Biochemistry, Biophysics and Biotechnology, Jagiellonian University, Gronostajowa 7, 30-387 Kraków, Poland

^4^Department of Molecular and Interfacial Biophysics, Faculty of Physics, Astronomy and Applied Computer Science, Jagiellonian University, Łojasiewicza 11, 30-348 Kraków, Poland

**Corresponding author*:** Małgorzata Lenartowicz, Department of Genetics and Evolution, Institute of Zoology and Biomedical Research, Jagiellonian University, Gronostajowa 9, 30-387 Kraków, Poland

E-mail address: [malgorzata.lenartowicz@uj.edu.pl](mailto:malgorzata.lenartowicz@uj.edu.pl)

Tel. +48 12 664 50 84

**Supplementary Material**

**Table S1. Antibodies used in immunoblotting**

| **Target protein** | **Primary Ab** | **Primary Ab dilution** | **Secondary Ab** | **Secondary Ab dilution** |
| --- | --- | --- | --- | --- |
| HO1 | Rabbit polyclonal, Enzo Life Sciences, #ADI-OSA-150-F | 1:3 000 | Goat anti-rabbit polyclonal, #A6154 (Sigma-Aldrich) | 1:20 000 |
| Slc48a1 (HRG1) | Rabbit polyclonal, Novus, #QC13349 | 1:500 | Goat anti-rabbit polyclonal, #A6154 (Sigma-Aldrich) | 1:20 000 |
| H-Ferritin | Rabbit polyclonal, kind gift from Dr. P. Santambrogio, San Raffaele Scientific Institute, Milan, Italy | 1:500 | Goat anti-rabbit polyclonal, #A6154 (Sigma-Aldrich) | 1:20 000 |
| Haptoglobin | Chicken polyclonal, US Biological | 1:10 000 | Rabbit anti-chicken polyclonal (#A9046 Sigma Aldrich) | 1:40 000 |
| Hemopexin | Mouse polyclonal, kind gift from E. Tolosano, University of Turin, Italy | 1:2 000 | Goat anti-mouse polyclonal, #A5278 (Sigma-Aldrich) | 1:20 000 |
| Lactate dehydrogenase 2 (LDH2) | Rabbit polyclonal, Proteintech, #14824-1-AP | 1:1000 | Goat anti-rabbit polyclonal, #A6154 (Sigma-Aldrich) | 1:20 000 |
| Albumin | Rabbit polyclonal, Santa Cruz Biotechnology, #SC- 50536 | 1:1 000 | Goat anti-rabbit polyclonal, #A6154 (Sigma) | 1:20 000 |
| Actin | Goat polyclonal, Santa Cruz Biotechnology, #SC-1615 | 1:2 000 | Donkey anti-goat polyclonal, #SC-2020 (Santa Cruz Biotechnology) | 1:20 000 |

**Table S2. Primers used in Real Time PCR analysis**

| **Target gene** | **Forward primer (5’→3’)** | **Reverse primer (5’→3’)** |
| --- | --- | --- |
| *Actb* | GGCCAACCGTGAAAAGATGACCCA | TACGACCAGAGGCATACAGGGACAG |
| *Hmox1* | AGAAGGGTCAGGTGTCCAGAGAAGGC | AGTGGGGCATAGACTGGGTTCTGCTT |
| *Hamp* | TGTCTCCTGCTTCTCCTCCT | CTCTGTAGTCTGTCTCATCTGTTG |

As a control, primers amplifying the housekeeping beta actin gene (*Actb*) were used. For RT-PCR analysis, standard curves were generated using serial dilutions of cDNA to determine the amplification efficiency of each primer pair.

**Table S3. Antibodies used in immunofluorescence analysis**

| **Target protein** | **Primary Ab** | **Primary Ab dilution** | **Secondary Ab/Fluorochrome** | | **Secondary Ab dilution** |
| --- | --- | --- | --- | --- | --- |
| HO1 | Rabbit polyclonal, Enzo Life Sciences, #ADI-OSA-150-F | 1:250 | Goat anti-rabbit conjugated with Cy3^®^ fluorochrome (Jackson Immunoresearch) | Goat Anti-Rabbit Cy3^®^3-conjugated AffiniPure Fab Fragment (Jackson Immunoresearch) | 1:500 for Goat anti-rabbit conjugated with Cy3 secondary antibodies and 1:50 for Fab fragments |
| Fpn | Rabbit polyclonal, Alpha Diagnostic, #MTP11-A | 1:250 | Goat anti-rabbit conjugated with Cy3^®^ fluorochrome (Jackson Immunoresearch) | | 1:500 |
| Slc48a1 (HRG1) | Rabbit polyclonal, kind gift from Prof. I. Hamza, University of Maryland, USA | 1:100 | Goat anti-rabbit conjugated with Cy3^®^ fluorochrome (Jackson Immunoresearch) | Goat Anti-Rabbit Cy3^®^3-conjugated AffiniPure Fab Fragment (Jackson Immunoresearch) | 1:500 for Goat anti-rabbit conjugated with Cy3 secondary antibodies and 1:50 for Fab fragments |
| TfR1 | Rabbit polyclonal, Santa Cruz Biotechnology, # SC-9099 | 1:50 | Goat anti-rabbit conjugated with Cy3^®^ fluorochrome (Jackson Immunoresearch) | | 1:500 |
| DMT1 | Rabbit polyclonal, Alpha Diagnostic,  #NRAMP24-A | 1:100 | Goat anti-rabbit conjugated with Cy3^®^ fluorochrome (Jackson Immunoresearch) | | 1:500 |
| Aquaporin 1 (AQP1) | Rabbit polyclonal, Alpha Diagnostic,  #AQP11-A | 1:200 | Goat anti-rabbit conjugated with Alexa488^®^ fluorochrome (Jackson Immunoresearch) | | 1:500 |
| F4/80 | Rat monoclonal, AbD Serotec,  # MCA497GA | 1:250 | Goat anti-rat conjugated with Alexa488® fluorochrome (Jackson Immunoresearch) | | 1:500 |
| Megalin | Rabbit polyclonal  Abcam #ab76969 | 1 : 250 | Goat anti-rabbit conjugated with Cy3^®^ fluorochrome (Jackson Immunoresearch) | | 1:500 |
| Cubilin | Sheep polyclonal  Novus #AF3700 | 1 : 100 | Donkey anti-sheep conjugated with Alexa488® fluorochrome (Jackson Immunoresearch) | | 1:500 |

**Supplementary Figure 1 – Uncropped Western Blot of hemopexin protein with loading control (albumin).**


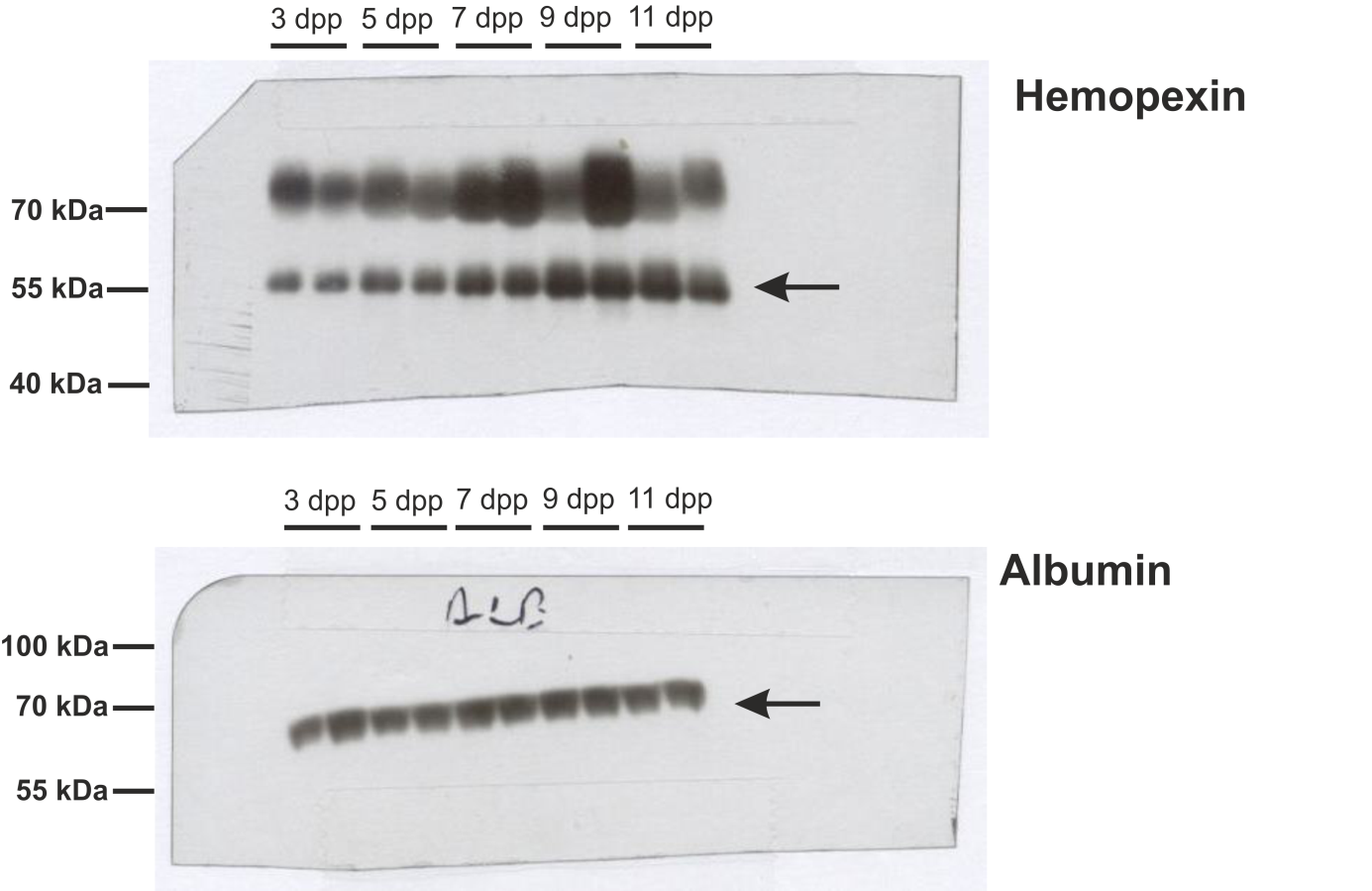


**Supplementary Figure 2 – Uncropped Western Blot of haptoglobin protein with loading control (albumin).**


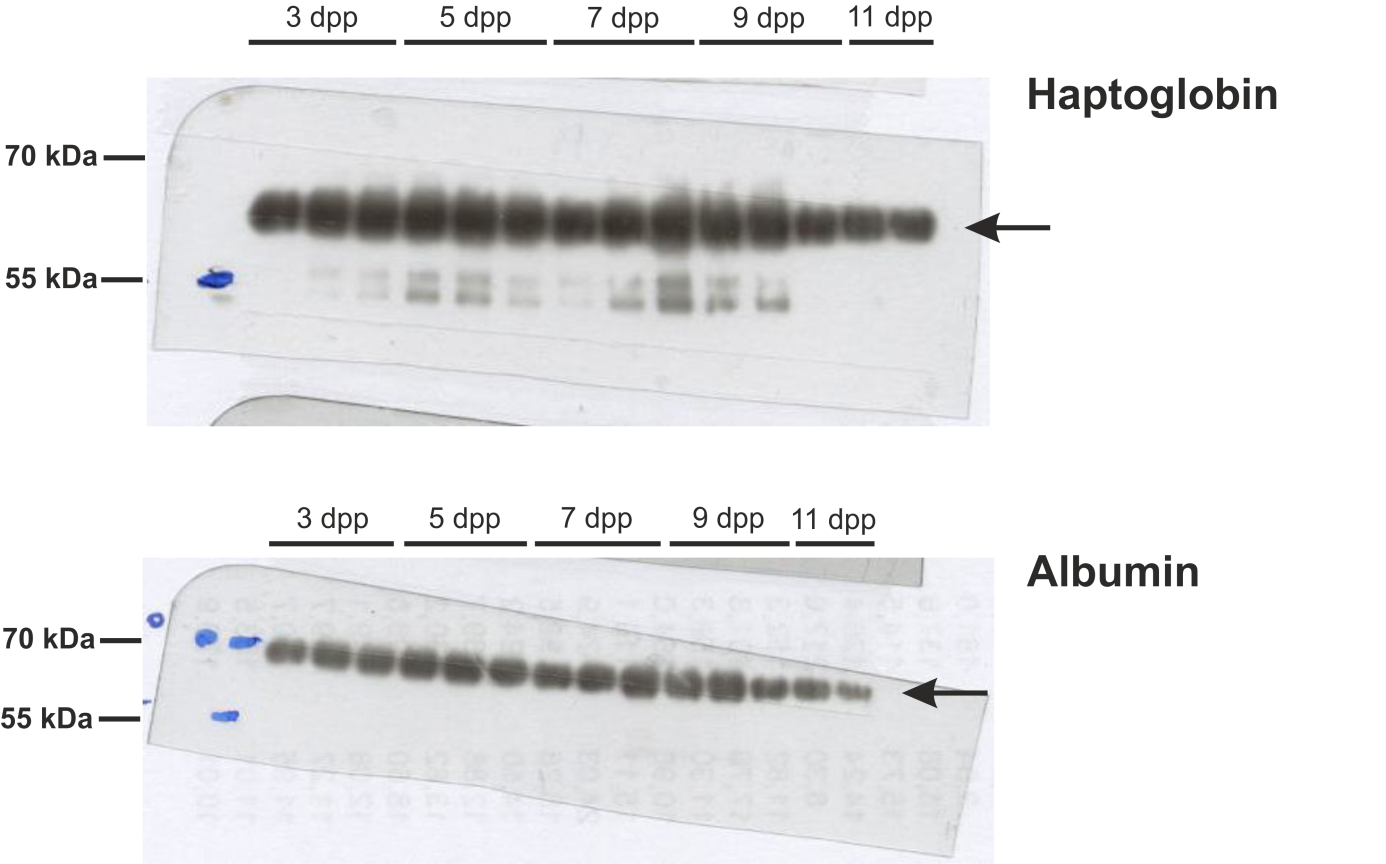


**Supplementary Figure 3 – Uncropped Western Blot of HRG1 (Slc48a1) protein with loading control (actin).**


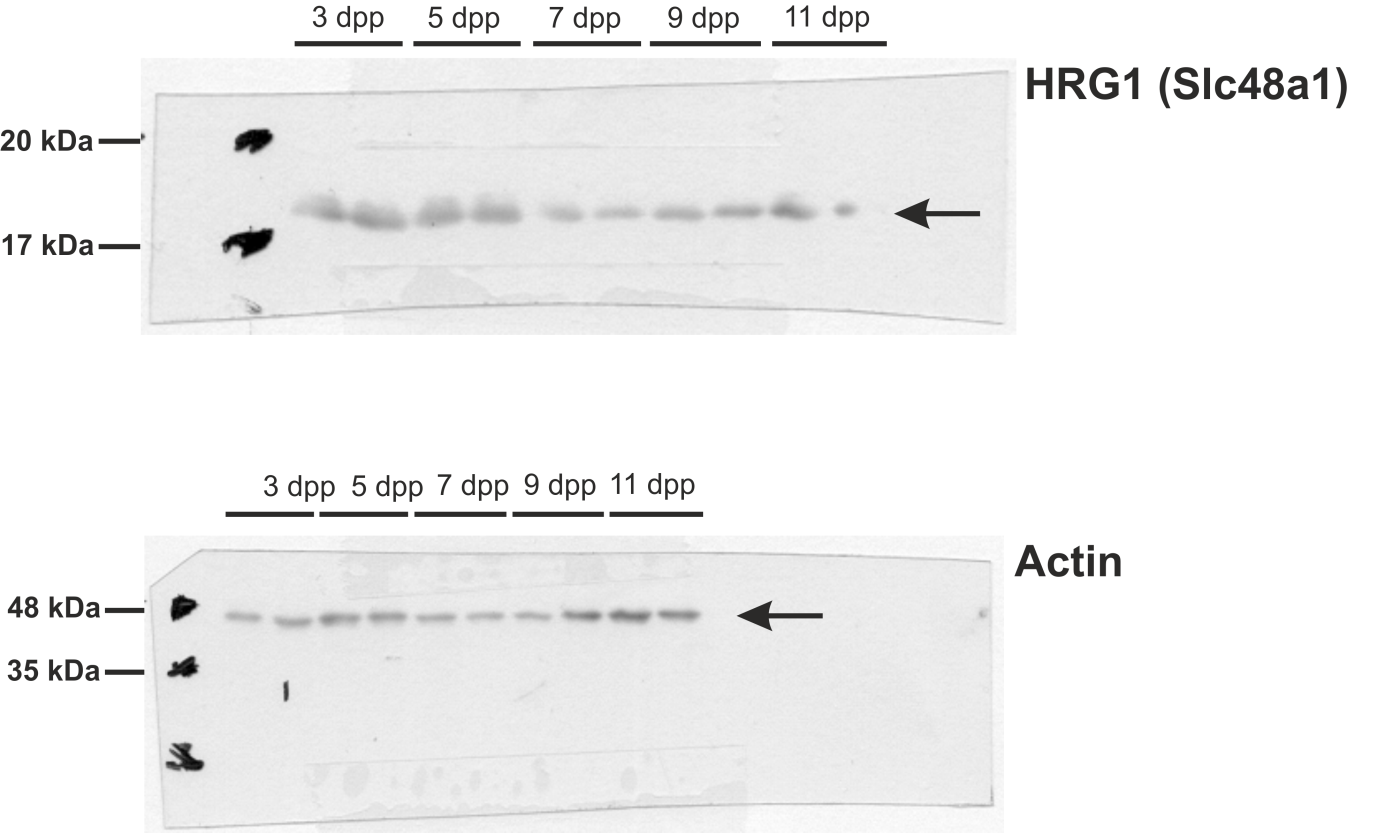


**Supplementary Figure 4 – Uncropped Western Blot of HO1 protein with loading control (actin).**


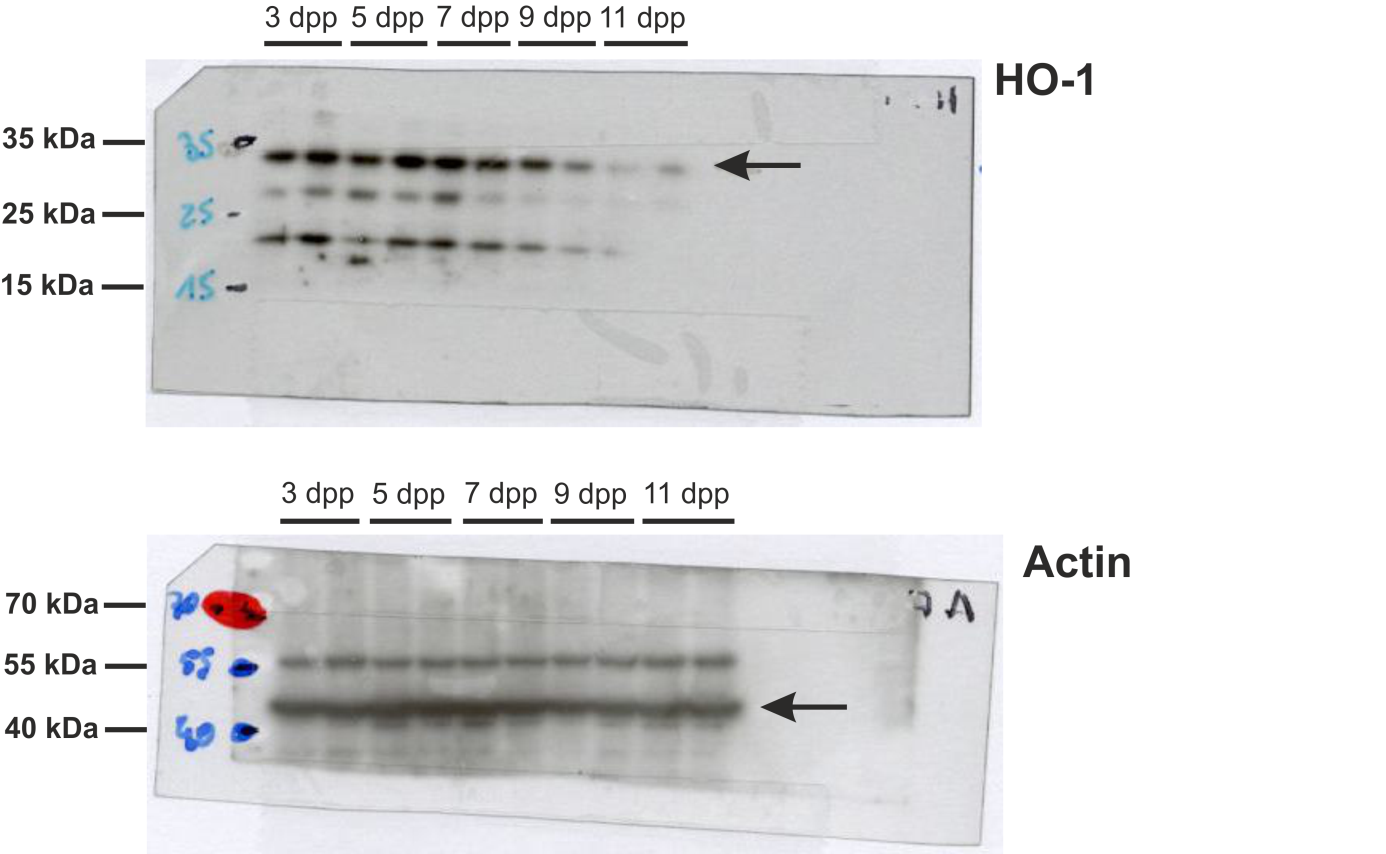


**Supplementary Figure 5 – Uncropped Western Blot of H-Ferritin protein with loading control (actin).**


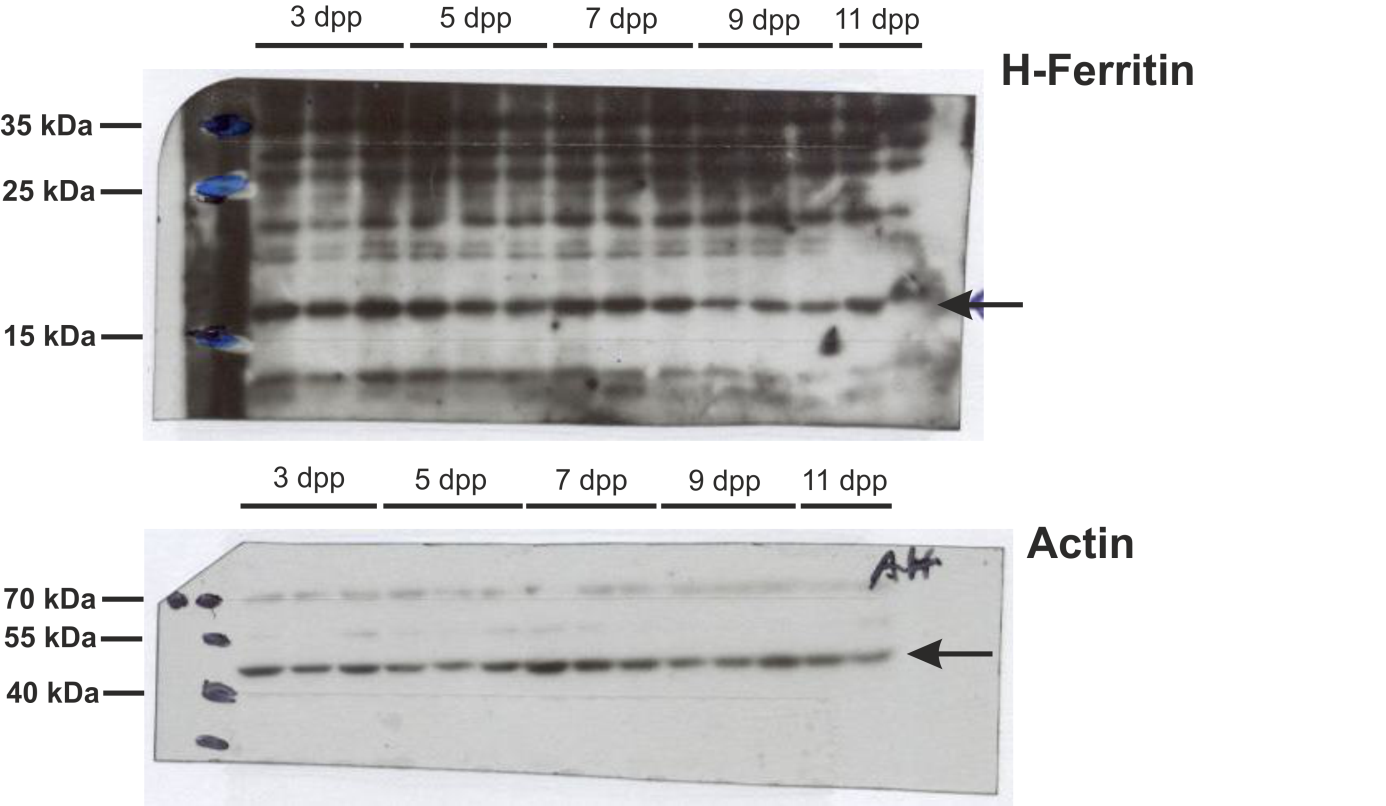

Supplement: Supplementary file 1 — Supplementary Materials [file 41598_2019_47414_MOESM1_ESM.docx]
